# Clinical analysis of cervical lymph node metastasis risk factors in patients with papillary thyroid microcarcinoma

**DOI:** 10.1007/s40618-018-0908-y

**Published:** 2018-06-06

**Authors:** Y. Luo, Y. Zhao, K. Chen, J. Shen, J. Shi, S. Lu, J. Lei, Z. Li, D. Luo

**Affiliations:** 1 West China School of Medicine, Sichuan University, Sichuan, China; 2 Department of General Surgery, Zhang jia-gang First People’s Hospital, Jiangsu, China; 3 Nanjing Medical University, Jiangsu, China; 4 Department of Surgical Oncology, Hangzhou First People’s Hospital, Nanjing Medical University, No. 261, Huansha Road, Shangcheng district, Hangzhou, 310006 Zhejiang China; 5 Zhejiang Chinese Medical University, Zhejiang, China; 6 Thyroid Surgery, West China Hospital of Sichuan University, Chengdu, China

**Keywords:** Papillary thyroid microcarcinoma, Lymph node metastasis, Neck dissection, Risk factors

## Abstract

**Purpose:**

Lymph node metastasis (LNM) is a vital prognosis factor in patients with papillary thyroid microcarcinoma (PTMC). The study tried to identify clinicopathological factors for LNM of PTMC.

**Methods:**

The clinicopathological data of 1031 patients with PTMC were extracted and analyzed. Univariate and multivariate analyses were used to identify risk factors associated with cervical lymph node metastasis. ROC analysis was used to determine the ideal critical points of the sum of the maximum diameter of multifocal in a unilateral lobe.

**Results:**

The probability of LNM, central lymph node metastasis (CLNM) and lateral lymph node metastasis(LLNM)of PTMC patients were 35.6, 33.7 and 5.6%, respectively. In addition, 1.9% PTMC had LLNM only. Male, age ≤ 40 years, tumor largest diameter ≥ 5 mm, multifocal, non-uniform echoic distribution, the sum of the maximum diameter of multifocal in a unilateral lobe ≥ 8.5 mm, tumors in the lower pole location were prone to CLNM. Ultrasound mix-echo, the sum of the maximum diameter of the multifocal ≥ 10.75 mm, tumors in the upper pole location were extremely prone to LLNM. T3 were prone to LLNM or skip LLNM.

**Conclusions:**

According to the clinicopathological characteristics of PTMC, the cervical lymph nodes should be correctly evaluated to guide the surgical treatment.

## Introduction

Papillary thyroid carcinoma (PTC) is the most frequent malignancy of the thyroid gland. The incidence of PTC rapidly increases worldwide in recent years and up to 43% of all these thyroid cancers are papillary thyroid microcarcinoma (PTMC) with a maximum diameter of 1.0 cm or less [1]. At present, it remains controversial whether PTMC needs surgery. Ito [2] does not recommend surgery for some PTMC patients. Sugitani et al. [3] suggest that the delayed surgery has no effect on outcomes. Similarly, the guideline of the American Thyroid Association (ATA) suggests that cautious observation is a safe and effective alternative to immediate surgical resection for PTMC [4]. However, PTC has a high metastasis rate especially central lymph node metastasis (CLNM), about 20 to 50% of PTC patients have CLNM [5]. Moreover, the CLNM can significantly affect the prognosis of thyroid cancer [5, 6]. PTMC is also potentially aggressive with a lymph node metastasis (LNM) rate of 37.3% [7]. The ultrasonic examination is important for the preoperative diagnosis of CLNM for PTMC patients, but this ultrasonic diagnosis can be difficult in some cases. We conducted this retrospective study of the clinicopathological features of PTMC to explore the lymph node metastasis of PTMC, and to provide evidence that is helpful for the clinical management of PTMC.

## Materials and methods

### General clinical materials

During the period from October 2010 to January 2017, 1031 cases of PTMC in the department of Surgical Oncology in Hangzhou First People’s Hospital were included in the study. Among these patients, 1010 cases were preoperatively diagnosed or suspected as PTMC by either fine needle aspiration(FNA) (518 cases) or B ultrasound (492 cases), and 21 cases were initially diagnosed as benign nodular goiter (19 cases) or hyperthyroidism (2 cases). All cases were confirmed to be PTMC successively by the intraoperative frozen and postoperative pathologic examination of resected tumor tissues. Criteria for admission: patients who had unilateral/bilateral PTMC in first surgery. Exclusion criteria: patients who had a history of other malignant tumors. This study was approved by the Ethics committee of our hospital and stayed in line with STROCSS criteria [8] with a unique identifying number (UIN: 3665) from the Research Registry. The data were analyzed in relation to gender, age at diagnosis, tumor largest diameter, capsular invasion, T staging, multifocal, the sum of the tumor diameter if multifocal, bilateral, tumor location (upper third, middle third, lower third and isthmus), B ultrasonography characteristics such as ultrasound intensity, composition, echoic distribution, tumor border, shape, calcification, aspect ratio, blood flow, and LNM that includes CLNM and lateral lymph node metastasis (LLNM). To evaluate the suspicious lateral lymph node metastasis, we used FNA for lymph node size larger than or equal to 1 cm and intraoperative frozen pathological examination for those smaller than 1 cm. The size and number of thyroid tumors, and the presence or absence of capsular invasion were recorded by high-frequency B ultrasonography. Each thyroid cancer patient was independently examined by two ultrasonographists. If two ultrasonographists had different opinions, the third ultrasonographist would participate in the examination to verify the data.

### Surgical procedures and Pathological examination

The PTMC patients in this study underwent open surgeries by a surgical team. The primary lesion was performed simultaneously with the cervical lymph node dissection. Ipsilateral lobe and isthmus resection was performed for unilateral primary lesions. Total thyroidectomy was performed for unilateral primary lesions requiring iodine 131 treatment. We generally performed the lymph node dissection of ipsilateral cervical central VI, which is recommended in China. The mean number of removed lymph nodes of the central compartment is 9.2. Lymph node dissections of contralateral cervical central VI and bilateral lateral compartments were performed only if there was lymph node metastasis in these compartments. As for all bilateral primary lesions, total thyroidectomy and bilateral cervical central VI lymph node dissection were performed, and lateral lymph node dissection was performed only for those having lateral lymph node metastasis. Additional lateral compartment neck dissections including levels II, III, IV and Vb would be performed if metastases were present in the lateral compartment. Level I and Va lymph nodes were spared unless there were clinically positive lymph nodes. Two expert pathologists independently examined the surgical specimens. Specifically, the histological type, number, largest diameter, and the presence/absence of capsular invasion in the primary thyroid tumor and the lymph node were determined.

### Follow-up

In the present study, each patient was followed up for more than a year. 92% patients (949 cases) were followed up from 15 to 90 months and none of them had distant metastasis. However, six patients suffered from local recurrence (one case of lateral compartment, and five cases of contralateral glands).

### Statistical analysis

The clinicopathologic features were compared using the Pearson Chi-square test. In multivariate analysis, the odds ratio (OR) and the 95% confidence interval (CI) for relationships between each significant variable and lymph node metastasis were calculated using backward stepwise binary logistic regression. *P* < 0.05 was considered to indicate a statistically significant difference. The receiver-operating characteristic (ROC) analysis was used to identify the cutoff point of the sum largest diameter of multifocal tumors for defining the risk of CLNM and LLNM. All analyses were performed using IBM SPSS, version 20.

## Results

### Clinicopathological characteristics of PTMC

There were 1031 patients with PTMC involved in this study. The mean age of these patients is 45.99 ± 10.94 yr (ranged from 14 to 74). 35.6% (367/1031) of cases exhibited neck lymph node metastasis including central compartment and lateral compartment. 33.7% (347/1031) had CLNM and 5.6% (58/1031) had LLNM. We further found that 38 patients (3.7%) had both CLNM and LLNM, the situation of which should be considered to be relatively severe. In addition, to our surprise, 1.9% (20/1031) presented only LLNM (Table 1).

**Table 1:** Number of neck lymph nodes

Neck lymph nodes	Metastasis	
	Negative	Positive
LNM	664 (64.4%)	367 (35.6%)
CLNM	684 (66.3%)	347 (33.7%)
LLNM	973 (94.4%)	58 (5.6%)
CLNM and LLNM	993 (96.4%)	38 (3.7%)
LLNM only	1011 (98.1%)	20 (1.9%)

The serum parathyroid hormone and calcium levels were assayed to assess the postoperative parathyroid function. There was no permanent hypoparathyroidism, which was defined as persistently low blood calcium levels (< 2 mmol/L) and subnormal parathyroid hormone levels by 6 months after surgery. The presence of recurrent laryngeal nerve (RLN) injury was determined by reviewing the surgical records and postoperative laryngoscopy reports. 16 patients presented transient RLN paralyses and recovered within 2–3 months of surgery. There was no permanent RLN paralysis. The morbidities of hypoparathyroidism and RLN injury in this study were lower than the literatures (permanent hypoparathyroidism:3.5–6.3%; permanent RLN injury: 1.3–3.6%) [9–12]. In the study, 0.39% (4/1031) patients exhibited pleura rupture (which was recognized during surgery and repaired), which is relatively higher than the literatures [13, 14]. The reason may be that there are more patients compared to the literatures undergoing dissections of lymph nodes posterior to the right recurrent laryngeal nerve. Some studies have reported that the incidence of chylous leakage in patients was from 0.5 to 8.3% [15–17]. In our study, 2.04% (21/1031) patients presented chylous leakage which resolved within 2–3 weeks of surgery. Horner’s syndrome, a very rare complication with less than 30 cases so far reported in the literature [18], was not observed in this study.

### Risk factors of LNM

First, we studied the relationship between these risk factors and LNM. And univariate analysis showed that LNM was significantly associated with tumor characteristics of age, gender, tumor largest diameter, capsular invasion, T staging, multifocality, bilateral, echoic distribution, calcification, and blood flow (Table 2). However, when these variables were included in multivariate analysis, bilateral was not a statistically significant predictor of the LNM. Some subgroups of the variables left were also excluded. Finally, male, young age (≤ 40 years), tumor largest diameter greater than 7 mm, T3, and multifocality were independent risk factors with odds ratios of 1.954, 1.916, 3.964, 1.924, and 1.858, respectively (Table 2).

**Table 2:** Univariate and multivariate analyses of LNM, including CLNM and LLNM, and clinicopathological characteristics of PTMC

Risk factors	LNM	Univariate analysis	Multivariate analysis		CLNM	Univariate analysis	Multivariate analysis		LLNM	Univariate analysis	Multivariate analysis	
		*P*	OR (95% CI)	*P*		*P*	OR (95% CI)	*P*		*P*	OR (95% CI)	*P*
Age, *n* (%)		< 0.001				< 0.001				0.384		
≤ 30	45 (49.5%)		2.103 (1.268–3.487)	0.004	42(46.20%)		1.969 (1.188–3.263)	0.009	7 (7.70%)			
30–40	100 (43.10%)		1.916 (1.328–2.763)	0.001	96 (41.40%)		1.888 (1.307–2.726)	0.001	16 (6.90%)			
40–50	109 (31.60%)		1.088 (0.775–1.525)	0.627	103 (29.90%)		1.067 (0.759–1.500)	0.709	14 (4.10%)			
> 50	113 (31.10%)		1		106 (29.20%)		1		21 (5.80%)			
Gender, *n* (%)		< 0.001		< 0.001		< 0.001		< 0.001		0.111		
Female	276 (32.70%)		1		260 (30.80%)		1		43 (5.10%)			
Male	91 (48.90%)		1.954 (1.387–2.752)		87 (46.80%)		1.961 (1.392–2.762)		15 (8.10%)			
Size, *n* (%)		< 0.001				< 0.001				<0.001		
≤ 2 mm	18 (19.10%)		1		16 (17.00%)		1		2 (2.10%)		1	
2–5 mm	138 (29.40%)		1.459 (0.831–2.56)	0.188	132 (28.10%)		1.586 (0.883–2.850)	0.123	14 (3.00%)		1.266 (0.281–5.710)	0.759
5–7 mm	83 (35.80%)		1.721 (0.945–3.131)	0.076	79 (34.10%)		1.917 (1.032–3.561)	0.039	14 (6.00%)		2.242 (0.494–10.185)	0.296
7–10 mm	128 (54.50%)		3.964 (2.188–7.181)	< 0.001	120 (51.10%)		4.236 (2.299–7.803)	< 0.001	28 (11.90%)		4.036 (0.920–17.705)	0.064
Capsular invasion, *n* (%)		0.016				0.021				< 0.001		
Negative	312 (34.30%)				297 (32.60%)				42 (4.60%)			
Positive	55 (45.50%)				50 (41.30%)				16 (13.20%)			
T staging, *n* (%)		0.003				0.029				<0.001		
T1	331 (34.30%)		1		315 (32.60%)				44 (4.60%)		1	
T3	33 (55.90%)		1.924 (1.086–3.408)	0.025	29 (49.20%)				12 (20.30%)		3.575 (1.692–7.552)	0.001
T4	3 (42.90%)		1.253 (0.26–6.035)	0.779	3 (42.90%)				2 (28.60%)		4.208 (0.702–25.223)	0.116
Multifocality, *n* (%)		< 0.001		< 0.001		< 0.001		< 0.001		0.092		
Negative	261 (32.50%)		1		243 (30.30%)		1		40 (5.00%)			
Positive	106 (46.50%)		1.858 (1.348–2.56)		104 (45.60%)		1.976 (1.434–2.724)		18 (7.90%)			
Bilateral, *n* (%)		0.007				0.002				0.172		
Negative	299 (33.90%)				280 (31.80%)				46 (5.20%)			
Positive	68 (45.30%)				67 (44.70%)				12 (8.00%)			
Ultrasound intensity, *n* (%)		0.385				0.417				< 0.001		
Hypo-echo	338 (35.30%)				320 (33.40%)				47 (4.90%)		1	
Moderate-echo	7 (30.40%)				6 (26.10%)				1 (4.30%)		0.842 (0.105–6.771)	0.871
Iso-echo	6 (37.50%)				6 (37.50%)				2 (12.50%)		3.387 (0.724–15.846)	0.121
High-echo	1 (20.00%)				1 (20.00%)				0 (0.00%)		–	–
Mix-echo	15 (51.70%)				14 (48.30%)				8 (27.60%)		4.729 (1.859–12.033)	0.001
Composition, n(%)		0.301				0.112				0.546		
Solid	365 (35.70%)				346 (33.90%)				57 (5.60%)			
Solid–cystic	2 (20.00%)				1 (10.00%)				1 (10.00%)			
Echo distribution, n(%)		0.004				0.003		0.038		0.905		
Uniform	108 (29.80%)				101 (27.80%)		1		20 (5.50%)			
Non-uniform	259 (38.80%)				246 (36.80%)		1.367 (1.018–1.835)		38 (5.70%)			
Border, *n* (%)		0.062				0.116				0.077		
Regular	101 (31.50%)				97 (30.20%)				12 (3.70%)			
Irregular	266 (37.50%)				250 (35.20%)				46 (6.50%)			
Shape, *n* (%)		0.232				0.37				0.045		
Regular	125 (33.20%)				120 (31.90%)				14 (3.70%)			
Irregular	242 (36.90%)				227 (34.70%)				44 (6.70%)			
Calcification, *n* (%)		0.016				0.016				0.075		
Negative	201 (32.20%)				189 (30.30%)				28 (4.50%)			
Fine	71 (39.20%)				68 (37.60%)				16 (8.80%)			
Thick	95 (42.04%)				90 (39.80%)				14 (6.20%)			
Aspect ratio, *n* (%)		0.19				0.17				0.548		
Proportioned	204 (33.90%)				192 (31.90%)				36 (6.00%)			
Disproportionality	163 (37.90%)				155 (36.00%)				22 (5.10%)			
Blood flow, *n* (%)		0.014				0.03				0.002		
Negative	192 (32.40%)				183 (30.90%)				22 (3.70%)			
Positive	175 (39.90%)				164 (37.40%)				36 (8.20%)			

### Risk factors of CLNM

After the integral analysis of LNM, we further studied the risk factors of CLNM. Univariate analysis showed that PTMC patients with CLNM were significantly associated with tumor characteristics of male, age, tumor largest diameter, capsular invasion, multifocality, bilateral, T staging, echoic distribution, calcification and blood flow (Table 2). However, when these variables were included in multivariate analysis, five factors were excluded. As a result, male, young age (≤ 40 years), tumor largest diameter greater than 5 mm, the multifocality, and non-uniform echoic distribution were independent risk factors with odds ratios of 1.961, 1.888, 1.917, 1.976, and 1.367 respectively (Table 2).

### Risk factors of LLNM

Univariate analysis showed that PTMC patients with LLNM were significantly associated with clinicopathological characteristics of tumor largest diameter, capsular invasion, T staging, ultrasound intensity, shape, and blood flow (Table 2). Nevertheless, after being included in multivariate analysis, tumor largest diameter, capsular invasion, ultrasound shape, and blood flow were excluded. T3 and ultrasound mix-echo were independent risk factors with odds ratios of 3.575, and 4.729, respectively (Table 2).

### LNM is related to the location of the gland in PTMC patients

In this study, there were 803 PTMC patients with single lesions in a unilateral lobe, and we divided the gland into four parts according to the upper pole, the middle pole, the lower pole, and the isthmus. The CLNM rates of the lesions in the upper pole, the middle pole, the lower pole, and the isthmus were 25.30, 29.40, 39.70, and 28.30%, respectively (*P* = 0.011). However, PTMC patients with lesions in the upper pole were most prone to LLNM, with a metastasis rate of 8.10% (*P* = 0.013). The probabilities of CLNM and LLNM in PTMC patients with lesions in the isthmus were 28.30 and 1.90%, respectively (Table 3).

**Table 3:** The risk of location in the solitary primary tumor for CLNM and LLNM

Location	CLNM		Metastasis rate (%)	*X* ^2^	*P*	Location	LLNM		Metastasis rate (%)	*X* ^2^	*P*
	No	Yes					No	Yes			
Upper	204	69	25.30	11.153	0.011	Upper	251	22	8.10	10.774	0.013
Middle	207	86	29.40			Middle	286	7	2.40		
Lower	111	73	39.70			Lower	174	10	5.40		
Isthmus	38	15	28.30			Isthmus	52	1	1.90		

### LNM is related to the multifocal in PTMC patients

**Fig. 1:**
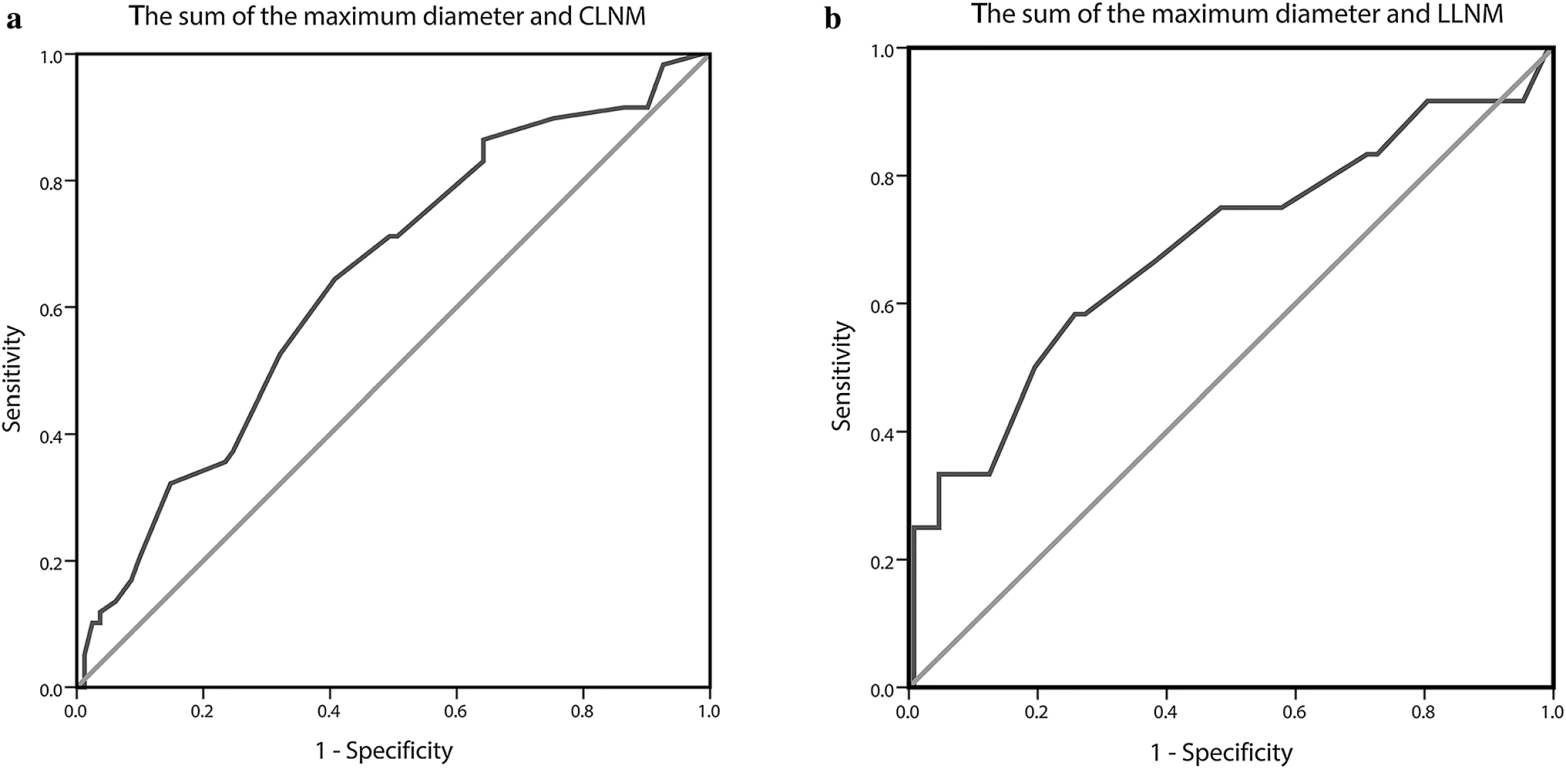
Outcomes of receiver-operating characteristic (ROC) curve analyses. **a** ROC curve analysis for the sum of the maximum diameter of multifocal in a unilateral lobe of CLNM: An index point ≥ 8.5 mm of the sum of the maximum diameter of multifocal was found to be the optimal point to distinguish between PTMC with and without CLNM. The sensitivity and specificity were 64.4 and 59.3%, respectively, with an AUC of 0.644. **b** ROC curve analysis for the sum of the maximum diameter of multifocal in a unilateral lobe of LLNM: An index point ≥ 10.75 mm of the sum of the maximum diameter of multifocal was found to be the optimal point to distinguish between PTMC with and without LLNM. The sensitivity and specificity were 58.3 and 74.2%, respectively, with an AUC of 0.687

In this study, there were 140 unilateral lobes with multifocal PTMC. We not only found that patients with multifocal PTMC were associated with CLNM, but also found that the sum of the maximum diameter of multifocal in a unilateral lobe was closely related to CLNM and LLNM. The ROC analysis showed that the areas under the ROC curves for the prediction of CLNM and LLNM by the risk factor sum ≥ 8.5 and ≥ 10.75 mm were 0.644, 0.687, respectively (Fig. 1a, b).

## Discussion

Generally most of PTMC has a good prognosis, but some PTMC patients can suffer from cancer death or poor quality of life. These poor outcome cases mainly occur in PTMC with metastatic lymph node or/and invasion of thyroid peripheral tissues, such as recurrent laryngeal nerve, internal jugular vein, trachea or esophagus. Compared with the intra-thyroid PTC group, the PTC group with LNM showed higher recurrence rates (5.2 vs. 31.5%), and higher disease-specific mortality (1.3 vs. 12.6%) [19]. In this study, no distant metastasis was found in all 1031 PTMC patients enrolled. Therefore, the distant metastasis is rare in PTMC and we focused on the association of LNM of PTMC patients with the clinicopathological characteristics and B ultrasonography characteristics. Our data analysis indicated a significant correlation between LNM and several risk factors.

Some studies used a tumor largest diameter of 5, 5.5, 7 mm to indicate the aggressiveness of PTMC [20–23]. In the present study, the tumor largest diameter ≥ 5 mm was an independent risk factor for CLNM. We suggested that patients with PTMC greater than 5 mm must be paid attention to the CLNM. Furthermore, the tumor largest diameter ≥ 7 mm was an independent risk factor for LNM. Therefore, for patients with PTMC larger than 7 mm, we should pay attention not only to CLNM, but also to LLNM.

The association of PTMC with gender and age remains controversial. Jeon et al. [24] showed that age ≥ 45 years was at a higher risk of LNM while male was not an independent risk factor. On the contrary, some studies revealed that both male and younger age were risk factors [21, 22, 25, 26]. In our study, male was an independent risk factor for LNM and CLNM, rather than for LLNM. We also found that age ≤ 40 years was an independent risk factor for LNM and CLNM, rather than for LLNM. These findings suggested that male and younger PTMC patients were prone to cervical LNM, especially CLNM (Table 2).

Additionally, we found that the multifocal lesion in PTMC was an important risk factor in CLNM. This finding is consistent with the data reported [7, 20, 21, 26–29]. Furthermore, with the increase of the sum of the maximum diameter of multifocal in a unilateral lobe, not only was the probability of CLNM increased, but also the probability of LLNM (Table 4). In the group with the sum of the maximum diameter > 8 mm, the rate of CLNM was 47.50%. The ROC analysis showed that the optimal point was 8.5 mm of the sum of the maximum diameter of multifocal. The sensitivity and specificity were 64.4 and 59.3%, respectively. In the group with the sum of the maximum diameter > 11 mm, the rate of LLNM was 19.40%. The ROC analysis showed that the optimal point was 10.75 mm of the sum of the maximum diameter of multifocal. The sensitivity and specificity were 58.3 and 74.2%, respectively (Fig. 1a, b). Therefore, we should pay more attention to the PTMC patients with multifocal lesion and their sum of the maximum diameter.

**Table 4:** The sum of the maximum diameters of all multifocal tumors

Sum of maximum diameters (mm)	CLNM		Metastasis rate (%)	*X* ^2^	*P*	LLNM		Metastasis rate (%)	*X* ^2^	*P*
	No	Yes				No	Yes			
≤ 8	48	21	30.40	9.011	0.011	66	3	4.30	6.229	0.044
8–11	21	19	47.50			37	3	7.50		
> 11	12	19	61.30			25	6	19.40		

In clinical practice, we used to observe a phenomenon with small tumor lesion but invaded extra thyroid tissues. There were 59 PTMC patients with T3 in our study. We found that T3 was associated with LLNM (*P* = 0.001, OR = 3.575), which was similar to Lu et al. [26] At the same time, we found 20 PTMC patients with only LLNM. Of these, four cases belonged to stage T3, and 16 cases were intra-thyroid lesions. The only LLNM rates of PTMC patients with T3 and with intra-thyroid lesion were 6.78% and 1.66%, respectively (*P* < 0.05), the former being 4.08 times more than the latter. The above results demonstrated that PTMC patients with T3 were prone to having LLNM or skip LLNM. LLNM shall be considered as a risk factor for distant metastatic spread and for cancer-related death [30]. Thus, we suggest that attention should be paid to the PTMC patients with T3 (Table 2).

The location of PTMC was related to the compartment of cervical lymph node metastasis [31]. We found that location in the lower third of the thyroid lobe conferred a higher risk for CLNM (*P* < 0.05), and that the location of upper third was related to a higher risk for LLNM (*P* < 0.05). However, the PTMC patients with lesions in the thyroid isthmus were prone to CLNM and rare to LLNM (Table 3). Thus, we should pay attention to estimate the compartment of cervical lymph node metastasis according to the location of the solitary primary tumor in the thyroid.

With the development of ultrasound technology, the diagnosis of PTMC has been improved. However, limited studies were performed to correlate LNM with ultrasound characteristics. A previous report [22] has reported that blood flow (present vs. absent) may improve the efficacy of predicting LNM (*P* < 0.001, OR = 5.3). In our study, blood flow was not a significant predictor of the LNM, CLNM or LLNM (*P* > 0.05). The present result indicated that PTMC patients with blood flow had limited value in predicting cervical LNM. But in this study, we found that the probability of CLNM of PTMC patients with non-uniform or uniform echoic distribution was significantly different (*P* = 0.003). It showed non-uniform echoic distribution was an independent predictive factor for CLNM (OR = 1.367). As for ultrasound intensity, we found that strong correlations of PTMC patients with mix-echo and LLNM (*P* < 0.01, OR = 4.729) (Table 2). These phenomenon suggested that PTMC patients with features of non-uniform echoic distribution and mix-echo should be paid attention to their respective the cervical CLNM and LLNM. In addition, other ultrasound features such as shape, composition, tumor border, calcification, and aspect ratio, were not associated with the cervical LNM.

There are some limitations in our study. First, the study is a single center study though it has a large sample size. Second, we did not divide the subtypes of PTMC so there may be some statistical bias. Third, it may omit few occult lymph node metastases because we did not perform lymph node dissection in patients not identified having contralateral lymph node metastasis. Finally, due to the difficulty in standardization of preoperative imaging diagnostic techniques of the lymph node assessment in the central compartment, the proportion of the suspicious lymph nodes in the central compartment was not calculated. Additional studies are required to address these issues.

## Conclusion

We found that the probability of cervical LNM was closely related to the clinical and pathological characteristics of PTMC. Male, age ≤ 40 years, tumor largest diameter ≥ 5 mm, multifocal, non-uniform echoic distribution, the sum of the maximum diameter of multifocal in a unilateral lobe ≥ 8.5 mm, tumors in the lower pole location were prone to CLNM. Ultrasound mix-echo, the sum of the maximum diameter of the multifocal ≥ 10.75 mm, tumors in the upper pole location were extremely prone to LLNM. Patients with T3 were prone to LLNM or skip LLNM. Therefore, according to the clinical and pathological characteristics of PTMC, the regional lymph nodes should be correctly evaluated to guide the surgical treatment of cervical LNM in PTMC.
